# Presenting the Uncertainties of Odds Ratios Using Empirical-Bayes Prediction Intervals

**DOI:** 10.1371/journal.pone.0032022

**Published:** 2012-02-21

**Authors:** Wan-Yu Lin, Wen-Chung Lee

**Affiliations:** 1 Institute of Epidemiology and Preventive Medicine, College of Public Health, National Taiwan University, Taipei, Taiwan; 2 Department of Biostatistics, University of Alabama at Birmingham, Birmingham, Alabama, United States of America; 3 Research Center for Genes, Environment and Human Health, National Taiwan University, Taipei, Taiwan; Vanderbilt University, United States of America

## Abstract

Quantifying exposure-disease associations is a central issue in epidemiology. Researchers of a study often present an odds ratio (or a logarithm of odds ratio, logOR) estimate together with its confidence interval (CI), for each exposure they examined. Here the authors advocate using the empirical-Bayes-based ‘prediction intervals’ (PIs) to bound the uncertainty of logORs. The PI approach is applicable to a panel of factors believed to be exchangeable (no extra information, other than the data itself, is available to distinguish some logORs from the others). The authors demonstrate its use in a genetic epidemiological study on age-related macular degeneration (AMD). The proposed PIs can enjoy straightforward probabilistic interpretations—a 95% PI has a probability of 0.95 to encompass the true value, and the expected number of true values that are being encompassed is 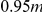 for a total of 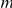 95% PIs. The PI approach is theoretically more efficient (producing shorter intervals) than the traditional CI approach. In the AMD data, the average efficiency gain is 51.2%. The PI approach is advocated to present the uncertainties of many logORs in a study, for its straightforward probabilistic interpretations and higher efficiency while maintaining the nominal coverage probability.

## Introduction

Quantifying the association between an exposure and a disease is a central issue in epidemiology. Although it has some limitations [1], [2], odds ratio (OR) is probably the most widely used measure of exposure-disease association in epidemiology. For a dichotomous exposure, OR is estimated by the famous formula: 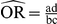, where a is the number of exposed cases; b, exposed controls; c, unexposed cases; and d, unexposed controls. To estimate the OR for an exposure with scale beyond simple yes/no, and simultaneously for multiple exposures, epidemiologists will need more than pencil and paper, but rather a logistic regression model to fit their data. Such a model fitting is usually performed by using statistical packages. The coefficients (except the intercept term) in a logistic regression model are the maximum likelihood estimates (MLEs) of the natural logarithms of odds ratios (logORs).

The ORs obtained from a study with a finite sample size are naturally subject to random variation to some degree, and therefore should not be taken as the true parameter values in and of themselves. To acknowledge this, epidemiologists often place a confidence interval (CI) around an OR estimate for each and every OR in the study. For a dichotomous exposure, the asymptotic 95% CI of logOR is simply: 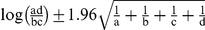. (The formula of the exact CI of a logOR can be found in Agresti [3].) For more general situations, the task of calculating CIs is again charged upon the logistic regression model.

Most epidemiologic studies are designed to simultaneously evaluate multiple risk factors. For example, with the advent of high-throughput technologies, genetic epidemiologists are often confronted with a large number of single nucleotide polymorphisms (SNPs), each calling for an OR (or a logOR) estimate and a CI. The total number of SNPs in a study can be in hundreds, thousands or even millions. Calculating CIs for so many logORs poses no special challenge. However, determining how to correctly interpret them often troubles even an experienced epidemiologist. Does the ‘confidence level’ associated with a CI signify the same meaning as ‘chance’ or ‘likelihood’ in our everyday usages, such that a 95% CI for a particular logOR has a probability of 0.95 to encompass the true logOR value? Unfortunately it does not. The confidence level of a CI actually relies on a ‘repeated-sampling’ interpretation. For a particular 95% CI, this means that if the procedure of producing it were to be repeated on multiple samples, the calculated 95% CIs (which would differ in location and in length for each sample) would encompass the true logOR value for 95% of the time [4]. This interpretation is based on sampling properties that are seldom realized in epidemiologic studies [2]. In fact, researchers in a study only get to work on one sample, the one in their study. It is cumbersome to have to conjure up multiple imaginary samples just to interpret a CI.

In this paper we advocate using the ‘prediction intervals’ (PIs) instead, to bound the uncertainty of the many logORs estimated in a study. The PI approach is applicable to a panel of factors believed to be exchangeable (that is, no extra information, other than the data itself, is available to distinguish some logORs from the others). For example, when we evaluate the associations of a panel of SNPs in a chromosomal region with a disease outcome, we may not distinguish certain SNPs from the others before seeing the data we have. Like conventional CIs, the calculation of PIs presents no special difficulty, involving just one extra step of simple arithmetic operations of the output of a logistic regression model. But unlike CIs, the proposed PIs can enjoy straightforward probabilistic interpretations; a 95% PI has a probability of 0.95 to encompass the true value, and the expected number of true values that are being encompassed is 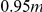 for a total of 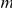 95% PIs. Furthermore, we will show that the proposed PI approach is theoretically more efficient (producing shorter intervals) than the traditional CI approach.

The PI approach is based on the well-known empirical Bayes (EB) principle [5]–[11]. (EB is a Bayesian approach, with the ‘prior information’ not to be supplied by the researchers, but estimated from the data itself.) Although the EB method has been developed for high-throughput data, most efforts are concentrated on microarray studies [12]–[18]; relatively few focus on (genetic) epidemiological studies [19]–[21]. In this paper, we extend the current EB methodologies in a number of ways. We allow for situations when the distribution of logORs is non-normal or unspecified, and then suggest the minimal number of factors (e.g., SNPs) to be analyzed together in a panel in order to guarantee the nominal coverage probability of a PI. To account for the phenomenon of linkage disequilibrium in genetic epidemiological studies, we also consider situations when the estimates of logORs are correlated with one another. The PI approach can enjoy straightforward probabilistic interpretations, and can achieve higher efficiency while maintaining nominal coverage probability.

## Methods

Suppose that the logistic regression model in a study involves a total of 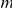 logORs (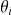, 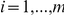). From the computer outputs, we obtain the point estimates (the regression coefficients), 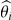, and their standard errors, 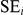, for 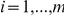. If desired, the variance-covariance matrix of the regression coefficients can also be produced, with the diagonal elements of the matrix being the variances, 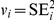 for 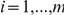, and the off-diagonal elements being the covariances, 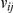 for 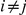. For the 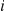
th logOR, the asymptotic 95% CI is 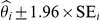. Note that the formulae for the CI and the following PI both require the asymptotic normality of MLEs. This may not hold true in studies with small sample sizes [22].

If these 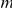 factors are ‘exchangeable’, the following PI approach can be employed. The condition of exchangeability means that no extra information, other than the data itself, is available to distinguish some 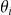
's from the others. For the 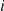
th logOR, the 95% PI is (see our Information S1 for the derivation):
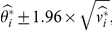
where 
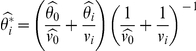
 (the posterior mean), 
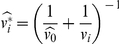
 (the posterior variance), 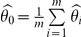 (the prior mean), and 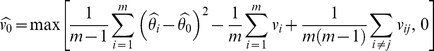 (the prior variance). From the formulae, we see that the individual posterior mean is a weighted average of the prior mean and the individual MLE, and that the prior mean is the average of all individual MLEs (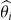
's). Compared to the individual MLEs, the individual posterior means are therefore shrunk toward the overall mean. As for the variances, we see that the posterior variance is a harmonic mean of the prior variance (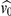) and the individual variance (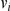). The posterior variance is thus guaranteed to be smaller than (with finite 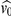) or equal to (with infinite 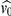) the individual variance. This suggests an efficiency gain through ‘borrowing strength’ [23] from one another in that panel of the 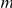 exchangeable factors.

Before turning to the ‘adjusted’ (or ‘model-based’) ORs, the researchers may often examine the ‘crude’ ORs one by one for each and every factor in the study (some refer to this as ‘univariate analysis’). The above PI formula can also be used for these crude ORs, except that now the covariances are not directly from a model output, but can be approximated using the formula: 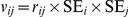, where the 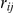 is the Pearson's correlation coefficient between the 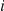
th and the 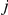
th factors, and the 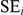 and 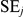, the standard errors of the 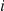
th and the 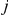
th crude logORs, respectively.

As the name suggests, a PI is a prediction of the whereabouts of the true value. A 95% PI is a probabilistic prediction implying that with a probability of 0.95, the true value is within the region it delimits. This probability statement can be directly applied to the very sample the researchers are working on. The next section will examine these properties in detail.

### Simulation Setup

We evaluate the performances of the PI approach in terms of average coverage probabilities. The average coverage probability for a total of 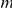 PIs is defined as:
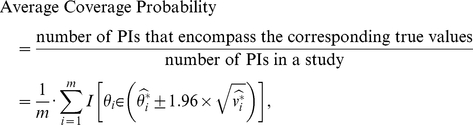
where 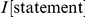, an indicator function, has a value of 1 if the statement is true, and a value of 0 if otherwise. The average coverage probability is also the expected proportion of PIs encompassing their corresponding true values. We study the situation when the true values of the logORs (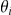
's) are normally distributed with a mean of 0 and a variance of 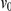. The 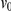 is studied for values of 1 (95% of the ORs are between 0.14 and 7.10), 0.5 (between 0.25 and 4.00) and 0.25 (between 0.38 and 2.66), respectively. We also study the situations of non-normal distributions. To do this, we deliberately set the distribution of 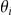
's to be very far from the normal: 40% of them coming from a beta distribution, 20% of them from a uniform distribution and the remaining 40% from a normal distribution. This mixture distribution is shown in our Information S1.

We also study situations when the estimates of the logORs (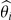
's) themselves are independent of one another, as well as when they are correlated. We assume two levels for the correlations: moderate correlation (with a third of the pair-wise correlations coming from a uniform [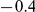, 0] distribution, and the remainder from a uniform [0, 0.4] distribution), and strong correlation (a third of the pair-wise correlations from a uniform [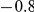, 0] distribution, and the remainder from a uniform [0, 0.8] distribution). The variances of the estimates of the logORs (the 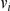
's) are studied for average values of 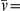
0.5 (uniform [0.25, 0.75] distribution; the ratio of upper limit to lower limit of 95% CI varies from 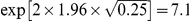 to 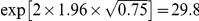), 0.25 (4.0 to 11.0) and 0.125 (2.7 to 5.5), respectively. 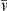 is related to the sample size (i.e., the number of subjects) of a study. Given a panel of factors, each with an exposure prevalence among controls drawn from a uniform [0.2, 0.8] distribution and 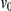 = 1, a 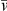 of 0.5, 0.25 or 0.125 approximately corresponds to a sample size of 50, 100 and 200, respectively. The simulation is performed 10,000 times for each scenario investigated.

## Results

### Simulation Results

**Figure 1:**
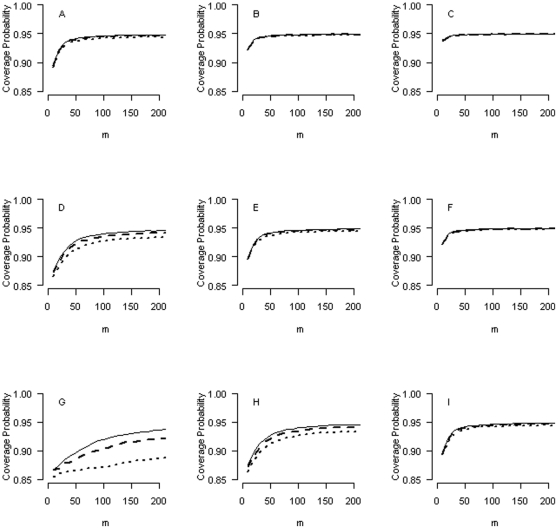
Coverage probabilities when different numbers of logORs (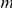) are considered for the prediction interval approach, assuming logORs are normally distributed. The different panels in the figure are arranged such that the 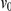 is 1, 0.5, and 0.25 (from top to bottom) and the 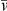 is 0.5, 0.25, and 0.125 (from left to right). Solid lines: independence among the estimates of the logORs; broken lines: moderate correlation among the estimates of the logORs; dotted lines: strong correlation among the estimates of the logORs.

**Figure 2:**
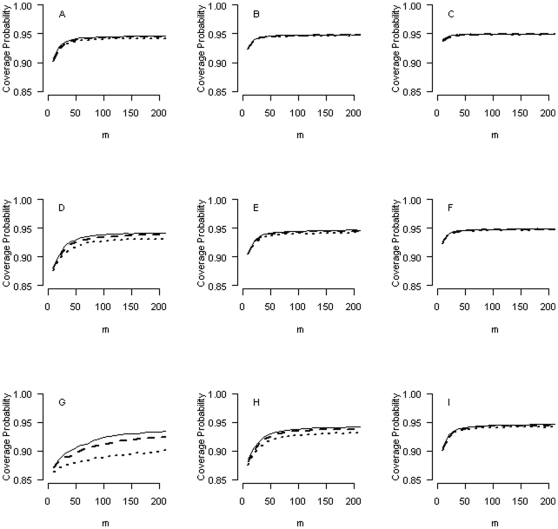
Coverage probabilities when different numbers of logORs (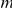) are considered for the prediction interval approach, assuming the distribution of logORs is non-normal. The different panels in the figure are arranged such that the 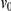 is 1, 0.5, and 0.25 (from top to bottom) and the 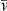 is 0.5, 0.25, and 0.125 (from left to right). Solid lines: independence among the estimates of the logORs; broken lines: moderate correlation among the estimates of the logORs; dotted lines: strong correlation among the estimates of the logORs.

Figure 1 plots the (average) coverage probability against 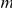, the number of logORs being considered for the PI approach, when 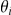
's are normally distributed (solid lines: independency; broken lines: moderate correlation; dotted lines: strong correlation). The different panels in the figure are arranged such that the 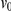 is 1, 0.5 and 0.25 (from top to bottom), and the 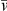 is 0.5, 0.25 and 0.125 (from left to right). We see that as more logORs are considered, their average coverage probability using the PI approach becomes closer to its nominal level of 95%. When 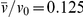 (panel C), 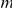 in the range of 10∼20 suffices to achieve the desirable coverage properties for the PI approach. With 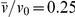 (panels B and F), 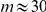 suffices. With 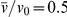, no more than 50 logORs are needed (panels A, E and I). For situations when 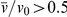, we need 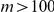 to guarantee the desired coverage probability (panels D, H and G). Figure 2 shows the coverage probabilities of the PI approach when the distribution of 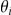
's is non-normal. We see that the results are very similar to those presented in Fig. 1.

**Figure 3:**
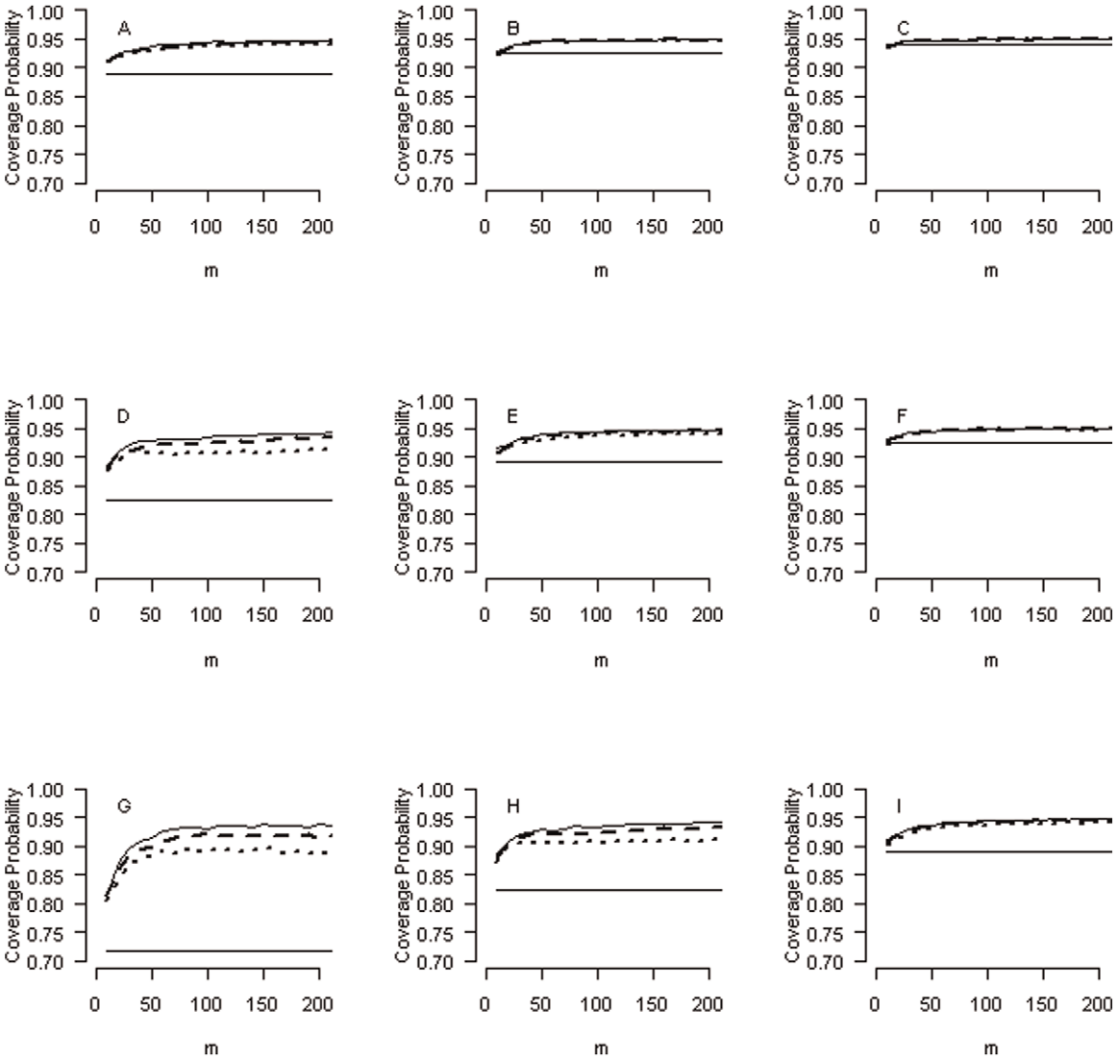
Coverage probabilities for those intervals that do not include zero (OR 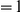, for the prediction interval approach (solid lines: independency; broken lines: moderate correlation; dotted lines: strong correlation) and for the confidence interval approach (constant solid lines), when logORs are normally distributed.

As more attention in a study is often given to those ORs that are significant. (Some will refer to these as the ‘discoveries’ or ‘findings’ of the study), we also examine the coverage probabilities for those intervals that do not include zero (OR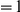). Figure 3 shows the results for the PI approach (solid lines: independency; broken lines: moderate correlation; dotted lines: strong correlation), and for the CI approach (constant solid lines), when 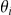
's are normally distributed. We see that even as we focus exclusively on those intervals that are ‘significant’, the intervals picked out by the PI approach can still guarantee the nominal coverage probability, that is, as the number of logORs is sufficiently large. Note that here we interpret the nominal coverage at its face value (95%, in our case) without resorting to multiple-testing adjustment of any kind. However, we see that the CI approach does not have this merit. In fact, significant intervals detected by the CI approach (without multiple-testing adjustment) can yield gross under-coverage (as low as 70%, in Fig. 3).

### Application to Real Data

We apply our PI approach to a genetic epidemiological study on age-related macular degeneration. Klein et al. [24] reported a data set containing 96 cases and 50 controls. We chose the SNPs within 1 Mb from the complement factor H gene on chromosome 1q32, which was reported as a promising region for age-related macular degeneration [25]. In this region (192,917,342 bp — 195,266,213 bp), 66 SNPs are informative (minor allele frequency
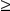
1%), and conform to the Hardy-Weinberg equilibrium (with the Hardy-Weinberg exact p values
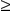
0.05 in the 50 controls).

A simple logistic regression model is fitted, one at a time, for each and every one of the 66 SNPs. At each SNP, the code is based on the number of copies of an arbitrarily chosen allele (0, 1 or 2). From the computer outputs, we obtain the point estimates (the regression coefficients), 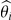, and their standard errors, 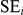, for the 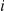
th logOR, for 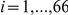. We also calculate the 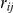, the Pearson's correlation coefficient between the 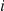
th and the 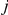
th SNPs. (The Pearson's correlation coefficients between allele counts (0, 1 or 2) at pairs of SNPs can measure the linkage disequilibrium between them [26]–[29].) Of the total 2145 pairs of SNPs, there are 1900 pairs with the absolute values of Pearson's correlation coefficients less than 0.2; 164 pairs between 0.2 and 0.4; 63 pairs between 0.4 and 0.8; and the remaining 18 pairs larger than 0.8. Using the formula presented in the Method section, we find 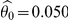, 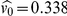, 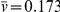 and 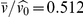. This example with a total of 66 SNPs is, therefore, amenable to the PI approach, since no more than 50 logORs are needed to guarantee the nominal coverage probability with 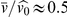 (see panels A, E and I in Figs. 1 and 2). If attention is to be restricted to those intervals that are significant, normality assumption will have to be invoked. Because 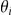
's are unknown, we perform the Shapiro-Wilk normality test on 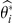
's in this example, and the p-value is 0.229.

**Figure 4:**
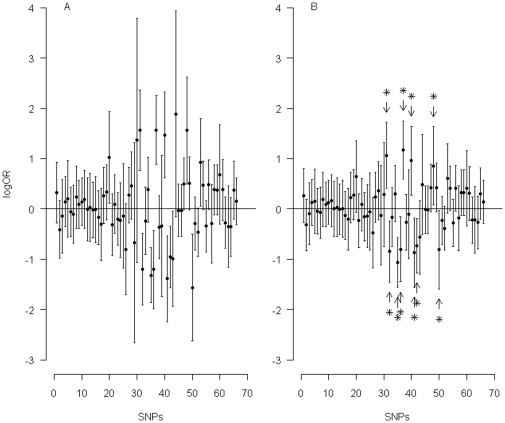
Results using the confidence interval approach and the prediction interval approach. (A) The 95% confidence intervals (CIs) and (B) the 95% prediction intervals (PIs) of the natural logarithms of odds ratios for the 66 single nucleotide polymorphisms (SNPs) in the age-related macular degeneration data. In (B), stars identify the 10 significant SNPs with corresponding 95% PIs not including zero.

Figure 4 shows the results using the CI approach (panel A), and the PI approach (panel B). The average length of the 95% CIs (for the logORs of the 66 SNPs) is 1.461, while the average length of the 95% PIs is 1.163, 20% shorter. Thus we see that the PI approach is statistically more efficient than the CI approach. Besides this, the PI approach has a straightforward probabilistic interpretation: within the regions delimited by the 66 PIs presented in the figure, one can expect to find 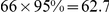 true logOR values. One can also focus on the PIs for the significant SNPs (a total of 10 SNPs, marked with stars in Fig. 4). Within the regions delimited by these 10 PIs, one can expect to find 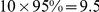 true logOR values. Since the regions do not contain any zeroes (true negatives), it means that the expected number of true positives among these 10 significant SNPs is 9.5.

## Discussion

The PI approach is based on the EB principle with a long history [5]–[11]. However, traditional CIs still dominate epidemiological studies. Here we advocate using PIs to bound the uncertainty of association measures for a panel of exchangeable factors. (The 66 SNPs in the previous example are indistinguishable from each other a priori.) The exchangeability condition allows us to make the assumption that the true logOR values (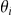
's) arise from a certain unknown/unspecified distribution, the key assumption of the PI approach. In this paper, we extend the current EB methodologies in the literature [23], [30]–[32] in a number of ways. These include: 1) we present a simple closed-form formula for the PI approach; therefore there is no need to perform computer iterations and/or complex modeling; 2) we allow for the situations when the distribution of 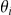
's is non-normal or unspecified, and then evaluate the required number of factors to guarantee the probabilistic interpretations for PIs (Parametric assumptions of 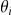
's have been commonly made in the literature, for example, normal [23], mixture normal [32] or ‘bell-shaped’ [30], [31].); and 3) we allow for situations when the estimates of 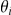
's are correlated with one another.

We need to emphasize that the PI approach is applicable to 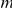 exchangeable factors, where 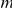 is a required number of factors (e.g., SNPs) to guarantee the probabilistic interpretations for PIs. The required 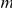 is 10∼20 when 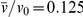, 30 when 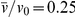, 50 when 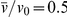 and 100+ when 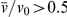. An applicable situation is to evaluate the associations of a panel of SNPs that are indistinguishable from one another a priori, as illustrated in our application. Other examples are panels of food items and occupational/environmental surveilance data, etc, where no extra information (other than the data itself) is available to distinguish some factors from others, and where the total number of factors investigated in a study is large enough for the PI approach. For example, a recent occupational surveillance on the New Zealand population was conducted to identify occupations that may contribute to the risk of non-Hodgkin's lymphoma [33]. Before the data analyses were conducted, a list of a priori high-risk occupations, including farmers, meat workers, painters, etc, was constructed based on previous literature. The EB method was then applied to the remaining hundreds of occupations having no a priori belief about their risks [33]. Also, we wish to point out that although we use the PI approach to bound the uncertainties of odds ratios in this paper, the methodologies can also be applied to bound the uncertainties of other association measures, such as risk ratios and risk difference, etc.

 When the PI approach is applicable to 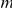 exchangeable factors (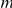 is large enough, as suggested above), it can provide not only a straightforward probabilistic interpretation, but also a more efficient interval inference than the CI approach. By ‘more efficient’, we mean smaller variances, and therefore shorter intervals while achieving the same probability to encompass the true value (though the confidence level of a CI relies on the ‘repeated-sampling’ interpretation). This efficiency gain comes from ‘borrowing strength’ [23] from one another in a panel of exchangeable factors. For the 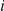
th 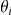, the efficiency gain of the PI approach to the CI approach is 
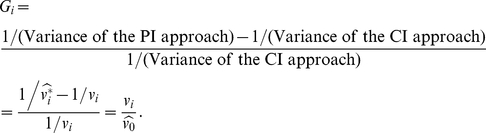
Because 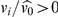, theoretically, the PI approach will be more efficient than the CI approach. For a study with a very large sample size (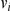
's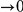), the efficiency gain by using the PI approach may be marginal (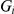
's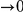). However for a moderate-sized study, the efficiency gain can be considerable. In Figs. 1 and 2, the average efficiency gains are, diagonally from left lower to right upper panels, 200%, 100%, 50%, 25% and 12.5%, respectively. In the example of the age-related macular degeneration data (Fig. 4), the average efficiency gain is 51.2%. In this paper, we focus on ‘interval estimates’ and show that the interval estimates of the EB-based PI approach have desirable statistical properties.

It is worth noting that the EB-based ‘point estimates’ are a different story: they have lower mean squared errors, but are biased by themselves [34]. Although the point estimates are not statistically unbiased, the EB-based adjustment can weed out false positives by shrinking imprecise outliers toward the overall mean. Through this shrinkage process, true positives may remain unambiguously positive, while false positives are likely to be removed (as shown by a large surveillance data set of occupation and cancer [35]). Using the EB-based adjustment in genetic studies is justifiable because follow-up or replication driven by false-positive findings often leads to a considerable cost.

The PI approach can provide a valid statistical inference for multiple tests. Treating multiple PIs as multiple tests, we show in the simulation that for those significant intervals identified by the PI approach (Fig. 3), the nominal coverage can be retained at its face value (95%, in our case) when 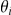
's are normally distributed. This suggests a link between the EB-based PI approach and the multiple-testing control of false discovery rate (FDR, defined as the expected proportion of false rejections among those that reject the null hypothesis of no association [36]). In fact, this connection follows directly from the Bayes theorem [13]. We apply Storey and Tibshirani's algorithm of FDR control [37] to the age-related macular degeneration data, and also identify the same 10 SNPs as detected by the PI approach. (The expected number of true positives among these 10 SNPs is 9.85, using Xie et al.'s permutation method [38].) More work needs to be done for multiple-testing controlling properties of the PI approach when the distribution of 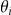
's is non-normal.

## Supporting Information

Information S1
**The derivation of the prediction interval approach, the distribution of** 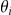 **and** 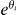 **for the non-normal situation, and** **Figure 3** **with** ***y*** **-axis ranging from 0.8 to 1.0.**
(DOC)
