# Supplementary material for: Presenting the Uncertainties of Odds Ratios Using Empirical-Bayes Prediction Intervals
Source: PLoS One. 2012 Feb 21;7(2):e32022. doi: 10.1371/journal.pone.0032022 (PMC3283699; doi:10.1371/journal.pone.0032022)
Supplement: Information S1 — The derivation of the prediction interval approach, the distribution of and for the non-normal situation, and Figure 3 with y -axis ranging from 0.8 to 1.0. (DOC) [file pone.0032022.s001.doc]

**Supporting Information S1 of**

**Presenting the Uncertainties of Odds Ratios Using Empirical-Bayes Prediction Intervals**

## Authors and Affiliations

### Wan-Yu Lin ^1,2^, Wen-Chung Lee ^1,3 §^

^1^ Institute of Epidemiology and Preventive Medicine, College of Public Health, National Taiwan University, Taipei, Taiwan.

^2^ Department of Biostatistics, University of Alabama at Birmingham, Birmingham, Alabama, U.S.A.

^3^ Research Center for Genes, Environment and Human Health, National Taiwan University, Taipei, Taiwan.

^§^Corresponding author

Correspondence & reprint requests: Prof. Wen-Chung Lee,

Rm. 536, No. 17, Xuzhou Rd., Taipei 100, Taiwan.

(FAX: 886-2-23511955; e-mail:wenchung@ntu.edu.tw)

## The derivation of the prediction interval approach

Assume that the true values of logORs ($\theta_{i}$, $i=1,...,m$) arise from a certain ‘prior’ distribution. (Alternatively if $m$ is large, we may posit that the ensemble of $\theta_{i}$’s constitutes a certain distribution and get rid of the term, ‘prior’.) We do not need to know the exact form of that distribution, apart from assuming its mean, $\theta_{0}$, and variance, $v_{0}$, to exist. Let $\widehat{\mathbf{\theta}}$ (an $m\times1$ vector with elements of $\widehat{\theta_{1}},...,\widehat{\theta_{m}}$) be a vector of maximum likelihood estimates (MLEs), and $\mathbf{V}$ (an $m\times m$ matrix with diagonal elements being variances, $v_{i}$ for $i=1,...,m$, and off-diagonal elements being covariances, $v_{ij}$ for $i\neq j$), its variance-covariance matrix, from a logistic regression analysis of a study. Standard MLE theory [39] implies that conditional on $\mathbf{\theta}$ (an $m\times1$ vector with elements of true values, $\theta_{1},...,\theta_{m}$), $\widehat{\mathbf{\theta}}$ is distributed as a multivariate normal distribution with a mean vector of $\mathbf{\theta}$ and a variance-covariance matrix of $\mathbf{V}$, asymptotically as the sample size tends to infinity, $n\to\infty$.

The unknown ‘prior’ mean and variance can be estimated using the method of moments. First, we note that

$$\begin{matrix} E\left(\sum_{i=1}^{m} \widehat{\theta_{i}}\right)=E\left(\mathbf{1}^{\mathbf{t}}\widehat{\mathbf{\theta}}\right) \\ \text{ }=\mathbf{1}^{\mathbf{t}}E(\widehat{\mathbf{\theta}}) \\ \text{ }=m\theta_{0}, \end{matrix}$$

where $\mathbf{1}$ is an $m\times1$ summing vector of 1’s. Therefore, an estimate of the prior mean is,

$\widehat{\theta_{0}}=\frac{1}{m}\sum_{i=1}^{m} \widehat{\theta_{i}}$.

Next, we note that

$$\begin{matrix} E\left[\sum_{i=1}^{m} \left(\widehat{\theta_{i}}-\widehat{\theta_{0}}\right)^{2}\right]=E\left(\widehat{\mathbf{\theta}}^{\mathbf{t}}\mathbf{C}\widehat{\mathbf{\theta}}\right) \\ \text{ }=\text{trace}\left[\mathbf{C}\text{ var(}\widehat{\mathbf{\theta}})\right]+E\text{(}\widehat{\mathbf{\theta}}^{\mathbf{t}})\text{ }\mathbf{C}\text{ }E\text{(}\widehat{\mathbf{\theta}}) \\ \text{ }=\text{trace}\left[\mathbf{C}\left(\mathbf{V}_{\mathbf{0}}+\mathbf{V}\right)\right] \\ \text{ }=(m-1)v_{0}+\left(1-\frac{1}{m}\right)\sum_{i=1}^{m} v_{i}-\frac{1}{m}\sum_{i\neq j} v_{ij}, \end{matrix}$$

where $\mathbf{C}$ is an $m\times m$ centering matrix with all its diagonal elements being $1-m^{-1}$, and all its off-diagonal elements being $-m^{-1}$, and $\mathbf{V}_{\mathbf{0}}$, an $m\times m$ matrix with diagonal elements being $v_{0}$’s and off-diagonal elements being 0’s. Therefore, an estimate of the prior variance is,

$$\widehat{v_{0}}=\max\left[\frac{1}{m-1}\sum_{i=1}^{m} \left(\widehat{\theta_{i}}-\widehat{\theta_{0}}\right)^{2}-\frac{1}{m}\sum_{i=1}^{m} v_{i}+\frac{1}{m\left(m-1\right)}\sum_{i\neq j} v_{ij},\text{ }0\right].$$

With $\widehat{\theta_{0}}$ and $\widehat{v_{0}}$ calculated, standard empirical Bayes theory [32] dictates that for a ‘normal-normal model’ (normal prior coupled with asymptotic-normal MLE),

$$\text{95\% PI (for }\theta_{i})=\widehat{\theta_{i}^{*}}\pm1.96\times\sqrt{\widehat{v_{i}^{*}}},$$

where $\widehat{\theta_{i}^{*}}=\left(\frac{\widehat{\theta_{0}}}{\widehat{v_{0}}}+\frac{\widehat{\theta_{i}}}{v_{i}}\right)\left(\frac{1}{\widehat{v_{0}}}+\frac{1}{v_{i}}\right)^{-1}$ and $\widehat{v_{i}^{*}}=\left(\frac{1}{\widehat{v_{0}}}+\frac{1}{v_{i}}\right)^{-1}.$ If the prior distribution is non-normal but the sample size of the study is large (to ensure the asymptotic normality of MLE), the prior information will be dominated by the data and will have little influence on the posterior distribution [40]. Therefore, the above PI formula still applies as long as the (normal) MLE ‘dominates’ the (non-normal) prior, i.e., as $\frac{v_{i}}{\widehat{v_{0}}}\to0$. Note that the PI approach proposed here is not applicable to studies with small sample sizes where the asymptotic normality of MLEs may not hold.

**References**

32. Qiu J, Hwang JT (2007) Sharp simultaneous confidence intervals for the means of selected populations with application to microarray data analysis. Biometrics 63: 767-776.

39. Andersen EB (1970) Asymptotic properties of conditional maximum-likelihood estimators. J R Statist Soc B 32: 283-301.

40. Rychlik I, Rydén J (2006) Probability and Risk Analysis. Ch.6 Introduction to Bayesian Inference. Springer.


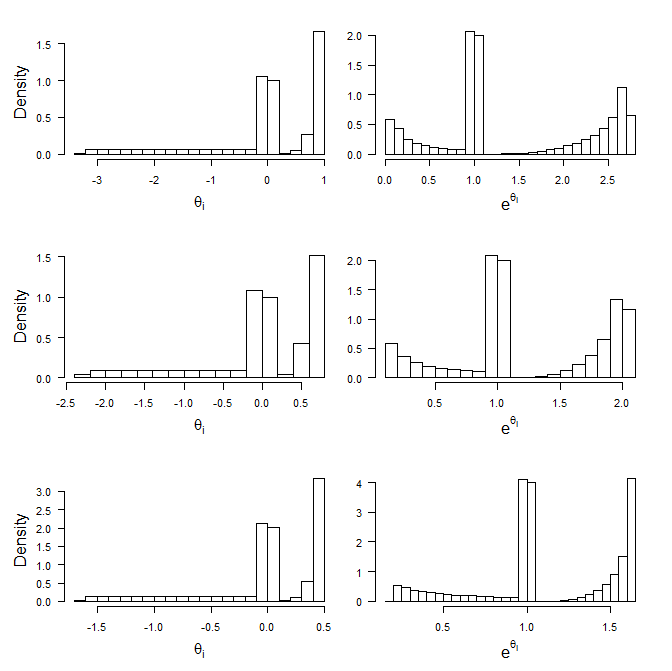


**The distribution of $\theta_{i}$ and $e^{\theta_{i}}$ for the non-normal situation**

The distribution of $\theta_{i}$ (left column) and $e^{\theta_{i}}$ (right column) for the non-normal situation. The $v_{0}$ is set at 1, 0.5, and 0.25 (from top to bottom). When $v_{0}$ is set at 1, we let 40% of $\theta_{i}$’s coming from a beta[5, 0.55] distribution, 20% of them, from a uniform[$-3.22$, 0] distribution, and the remaining 40%, from a normal[0, $10^{-4}$] distribution. When $v_{0}$ is set at 0.5 or 0.25, the distribution of $\theta_{i}$ is rescaled to meet the setting of $v_{0}$ while maintaining the same distribution prototype.


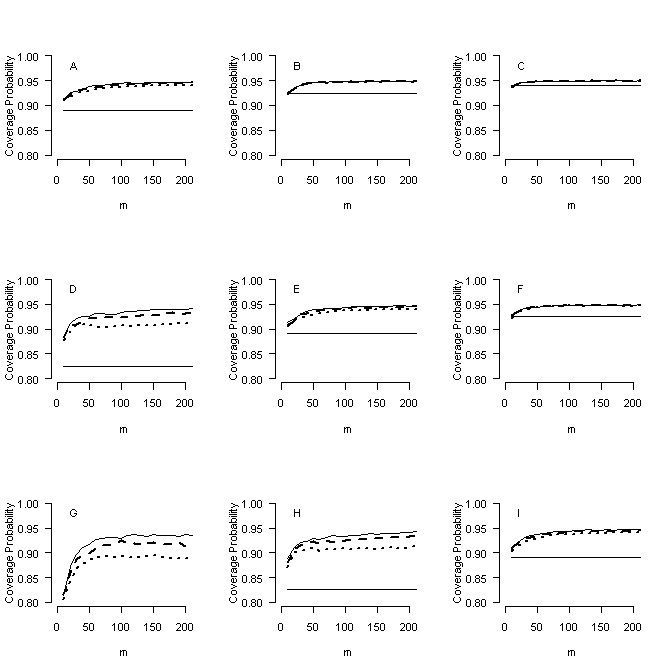


**Figure 3 with *y*-axis ranging from 0.8 to 1.0**

Coverage probabilities for those intervals that do not include zero (OR$=1$), for the prediction interval approach (solid lines: independency; broken lines: moderate correlation; dotted lines: strong correlation) and for the confidence interval approach (constant solid lines), when $\theta_{i}$’s are normally distributed.
